# Cluster subcutaneous allergen immunotherapy as a sustainable practice towards net zero healthcare

**DOI:** 10.1002/clt2.12241

**Published:** 2023-07-03

**Authors:** Alicia Domínguez Estirado, Inmaculada Sánchez‐Machín, Paloma Poza‐Guedes, Ruperto González‐Pérez

**Affiliations:** ^1^ Allergy Department Hospital Universitario Gregorio Marañón Madrid Spain; ^2^ Allergy Department Hospital Universitario de Canarias Tenerife Spain; ^3^ Allergen Immunotherapy Unit Hospital Universitario de Canarias Tenerife Spain; ^4^ Severe Asthma Unit Hospital Universitario de Canarias Tenerife Spain

To the Editor,

As the health care sector accounts for approximately 5% of global greenhouse‐gases (GHG) emissions, several health systems are calling for adoption of transparent and standardized metrics for GHG accounting, paving the way towards net zero healthcare.^1^ Although allergen immunotherapy (AIT) has been proposed as a prototype of individualized medicine in terms of clinical response and safety for allergic disease, commuting to medical facilities and lengthy build‐up schedules have been described as limiting factors to treatment compliance among subjects on subcutaneous immunotherapy (SCIT).^2^ Cluster AIT schedules are variations of conventional AIT regimes, in which the timeframe from induction to maintenance phase is much shorter compared to conventional AIT.^3^ Hence, we assessed the contribution of a SCIT cluster scheduled intervention in the reduction of the carbon footprint in subjects starting SCIT.

In this single‐center retrospective analysis, sociodemographic data, clinical profile, the SCIT dosing schedule and the number of required physical visits, and road travelled distance to our Institution during the build‐up phase of SCIT was collected from patients' electronic medical records from November 2021 to January 2022. Following routine clinical practise, only subjects with a confirmed Allergist SCIT‐prescription, following a positive skin prick test and/or a specific IgE to a corresponding panel of standardized aeroallergen were included.^4^ The investigation was approved by the local Ethical Committee (institutional code Complejo Hospitalario Universitario de Canarias 2022‐13, on 2022, February 24^th^) and funded by Fundación Canaria Instituto de Investigación Sanitaria de Canarias, Servicio Canario de Salud, grant number OA17/042. The authors declare no conflict of interest and the funders had no role in the design of the study; in the collection, analyses, or interpretation of data; in the writing of the manuscript, or in the decision to publish the results.

A total of 710 doses of SCIT were administered in 145 patients during the 12‐week study period (Table 1). All subjects successfully completed a SCIT cluster protocol, including the administration of 2 injections at a 30‐min interval in weekly visits to reach the maintenance dose in 1–4 weeks. A total of 97 out of 145 subjects (66.8%) completed a cluster SCIT schedule with allergoids (adsorbed to aluminium hydroxide or L‐tyrosine and Glutaraldehyde‐modified extracts), while 48 patients (33.2%) followed a SCIT cluster regime with solely adsorbed to aluminium hydroxide extracts. Regarding the composition of the prescribed SCIT, the combination of House Dust Mites (HDM) was most frequently prescribed in 64 out of 145 patients (44.13%), followed by HDM and *Blomia tropicalis* in 50 subjects (34.48%). The mean number of required physical visits per patient following a cluster schedule was significantly (<0.001) reduced compared to a conventional SCIT regime. The overall road travelled distance was 16,613 km for all 145 subjects completing a cluster SCIT schedule, in contrast to a total of 31,808 km following a conventional SCIT regime. In addition, the estimated annual carbon footprint for the cluster schedule was 8960 kg Co2e with a potential reduction of 8204 kg Co2e related to a conventional SCIT regime (Figure 1).^5^ Ten adverse events (AE) related to the cluster SCIT schedule were reported in 6 out of 145 patients (4.13%), increasing the mean (SD) number of outpatients extra‐visits to the AIT Unit (AIU) by 1.5 ± 1.2 times to reach the cluster SCIT maintenance dose. Nine out these 10 AEs were described as mild non‐immediate local reactions after subcutaneous injection. The remaining AE was considered a Grade‐II late moderate reaction, successfully treated at home with the regular patient's medication after a telephone consultation to the AIU.^6^

**TABLE 1:** Demographic and clinical characteristics of subjects.

Demographic and clinical characteristics	Total (*n* = 145)
Age (y.o., mean ± SD)	43.5 ± 14.6
Gender (%)	
Female	73 (50.34)
Male	72 (49.76)
Allergy history (%)	
Atopic eczema	34 (23.44)
Food allergy	20 (13.7)
Physical (cold) urticaria	4 (2.7)
Drug allergy	3 (2)
Chronic spontaneous urticaria	2 (1.3)
Eosinophilic oesophagitis	1 (0.6)
Atopic condition treated with immunotherapy (%)	
Rhinitis	20 (13.7)
Conjunctivitis	2 (1.3)
Rhino‐conjunctivitis	54 (37.2)
Asthma	0 (0)
Asthma and rhino‐conjunctivitis	69 (47.5)

**FIGURE 1:**
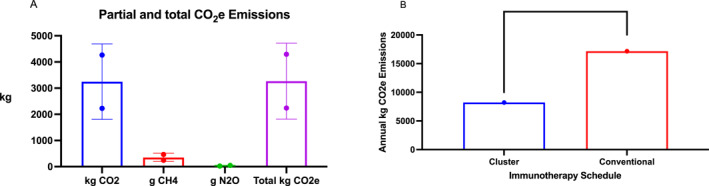
(A) Contribution of cluster and conventional allergen immunotherapy (AIT) schedules to partial (CO_2_, CH_4_, and NO_2_) and total (CO_2_e) carbon footprint emissions from 2021 November to 2022 January. (B) Annual CO_2_e of cluster and conventional AIT schedules. Asterisks indicate statistical signiﬁcance (****p < 0.001).

To our knowledge, this is the first study to investigate the contribution of a cluster SCIT schedule in the reduction of the carbon footprint related to scope 1 emissions as a part of our shared responsibility to decarbonize (Figure 2). Health care professionals are called to participate and implement sustainable medical practice principles to routinely clinical practice. Cluster allergen SCIT may contribute to reduce direct emissions from healthcare facilities to achieve carbon neutrality.

**FIGURE 2:**
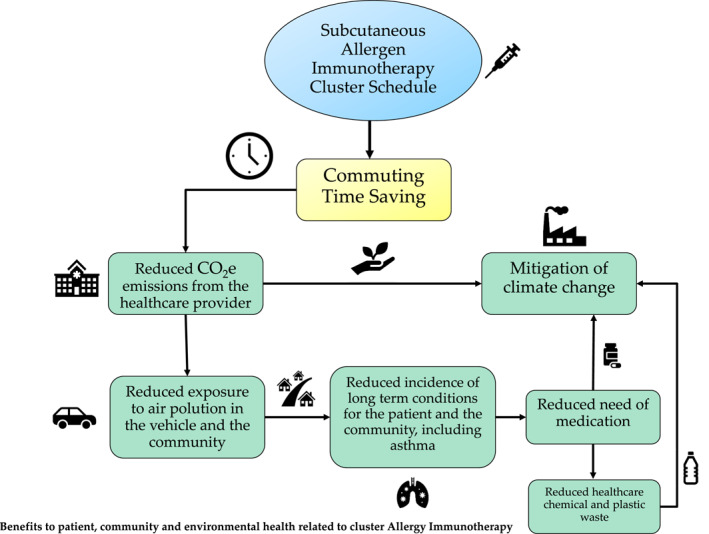
Potential benefits to patient, community and environmental health related to cluster scheduled subcutaneous immunotherapy (SCIT).

## AUTHOR CONTRIBUTIONS

**Alicia Domínguez Estirado**: Data curation (Equal); Investigation (Equal); Validation (Equal); Writing – original draft (Equal); Writing – review & editing (Equal). **Inmaculada Sánchez‐Machín**: Data curation (Equal); Formal analysis (Equal); Methodology (Equal); Resources (Equal); Supervision (Equal); Validation (Equal); Visualization (Equal). **Paloma Poza‐Guedes**: Data curation (Equal); Formal analysis (Equal); Investigation (Equal); Methodology (Equal); Software (Equal); Supervision (Equal); Validation (Equal); Visualization (Equal); Writing – original draft (Equal). **Ruperto Gonzalez Perez**: Conceptualization (Equal); Data curation (Equal); Formal analysis (Equal); Funding acquisition (Equal); Validation (Equal); Writing – original draft (Equal).

## FUNDING INFORMATION

Fundación Canaria Instituto de Investigación Sanitaria de Canarias (FIISC)

## Data Availability

The data that support the findings of this study are available from Servicio Canario de Salud but restrictions apply to the availability of these data, which were used under license for the current study, and so are not publicly available. Data are however available from the authors upon rea‐sonable request and with permission of Servicio Canario de Salud.
